# Comparison of nucleotide sequences of recent and previous lineages of peste-des-petits-ruminants viruses of sheep and goats in Nigeria

**DOI:** 10.4102/ojvr.v83i1.1163

**Published:** 2016-08-31

**Authors:** Samuel Mantip, Melvyn Quan, David Shamaki, Moritz van Vuuren

**Affiliations:** 1 Department of Veterinary Tropical Diseases, University of Pretoria, South Africa; 2 Viral Research Division, National Veterinary Research Institute, South Africa

## Abstract

Peste-des-petits-ruminants virus (PPRV) is a highly contagious, fatal and economically important viral disease of small ruminants that is still endemic and militates against the production of sheep and goats in endemic areas of the world. The aim of this study was to describe the viral strains within the country. This was carried out by collecting tissue and swab samples from sheep and goats in various agro-ecological zones of Nigeria. The phylogeny of archived PPRV strains or isolates and those circulating and causing recent outbreaks was determined by sequencing of the nucleoprotein (N)-gene. Twenty tissue and swab samples from apparently healthy and sick sheep and goats were collected randomly from 18 states, namely 3 states in each of the 6 agro-ecological zones visited. A total of 360 samples were collected. A total of 35 samples of 360 (9.7%) tested positive by reverse transcriptase–polymerase chain reaction, of which 25 were from oculo-nasal swabs and 10 were from tissue samples. Neighbour-joining phylogenetic analysis using Phylogenetic Analysis Using Parsimony (PAUP) identified four different lineages, that is, lineages I, II, III and IV. Interestingly, the Nigerian strains described in this study grouped in two separate major lineages, that is, lineages II and IV. Strains from Sokoto, Oyo, Plateau and Ondo states grouped according to the historical distribution of PPRV together with the Nigerian 75/1 strain of lineage II, while other strains from Sokoto, Oyo, Plateau, Akwa-Ibom, Adamawa, Kaduna, Lagos, Bauchi, Niger and Kano states grouped together with the East African and Asian strains of lineage IV. This finding confirms that both lineage II and IV strains of PPRV are circulating in Nigeria. Previously, only strains of lineage II were found to be present in the country.

## Introduction

Peste-des-petits-ruminants virus (PPRV) belongs to the family *Paramyxoviridae* and genus *Morbillivirus*. PPRV is a negative-sense, single-stranded, non-segmented Ribonucleic Acid (RNA) virus. It is enveloped, pleomorphic, and ranges in size from 150 nm to 700 nm. Six transcriptional units encode eight proteins; the nucleocapsid protein (N), phosphoprotein (P), large polymerase (L), fusion (F), haemagglutinin (H) and the matrix (M) protein and non-structural proteins C and V.

Peste-des-petits ruminants (PPR) has been mistaken for rinderpest since the fourth century AD (Curasson 1932; Henning 1956). In 1942, Gargadennec and Lalanne reported a disease in sheep and goats that resembled rinderpest in the West African country of Ivory Coast. Clinical observations of PPR differed to rinderpest, infected sheep and goats were unable to transmit the disease to in-contact cattle (Gargadennec & Lalanne 1942). The first reported isolation of the aetiological agent of the disease was in 1962 by Gilbert and Monier. In Nigeria, PPRV was successfully isolated in 1975 (Taylor 1979).

PPRV is distributed widely across West, Central, East and some parts of North Africa, the Arabic Peninsula, the Middle East and Asia (Nanda *et al*. 1996). The PPR-endemic areas of the world are also important sheep- and goat-rearing regions, and small livestock serve as an important means of providing food and commodities for trade. The production of these economically important animals for poor rural farmers is seriously hampered by the high morbidity caused by PPRV. Mortality from PPRV in a naive flock can be as high as 80% – 90%. PPR manifests with pyrexia, oculo-nasal mucopurulent discharges, stomatitis, enteritis and conjunctivitis (Losos *et al*. 1986). In severe cases, death is caused by secondary bacterial pneumonia or severe dehydration caused by acute diarrhoea. Abortion caused by co-infection of both PPRV and pestiviruses has been reported (Abubakar, Ali & Khan 2008). Because of the highly contagious nature of the disease, it can be transmitted easily to healthy animals through direct contact with the secretions and excretions of infected animals (Ezeibe *et al*. 2008). The virulence of PPRV varies from strain to strain, although only one serotype is recognised.

PPRV is sometimes referred to as a more serious disease of goats than sheep; however, outbreaks affecting both sheep and goat populations have been reported (Chauhan *et al*. 2009; Roeder *et al*. 1994; Taylor & Abegunde 1979; Taylor *et al*. 2002; Singh *et al*. 2004; Wang *et al*. 2009). In some outbreaks, goats appear not to be affected, while sheep succumb with high rates of morbidity and mortality (Yesilbag *et al*. 2005). The reason for this variability is unclear, but both viral strains and host species are thought to be of importance. Different strains of PPRV exhibit varied virulence when experimentally infected into the same breed of goat (Couacy-Hymann *et al*. 2007), and different breeds of goats have been shown to respond differently to infection with the same virus (Diop, Sarr & Libeau 2005).

The current molecular characterisation of PPRV strains based on the N-gene divides them into four genetically distinct lineages (Banyard *et al*. 2010). Lineage I is represented mainly by West African strains from the 1970s and recent strains from Central Africa; lineage II by West African strains from the Ivory Coast, Guinea and Burkina Faso; lineage III by isolates from Eastern Africa, Sudan, Yemen and Oman; lineage IV includes all viruses isolated from recent outbreaks across the Arabian Peninsula, the Middle East and southern Asia (and recently across several African territories (Banyard *et al*. 2010).

Because of the adverse consequences posed by PPRV in the production of sheep and goats in Nigeria, there is a need to undertake a study of PPRV in sheep and goats from recent outbreaks across the different regions of the country. It is important to understand the spread of the pathogen within the country in comparison with other endemic areas of the world. The sequences derived from this study will provide the basis for better understanding of the epidemiology of PPRV.

The aim of this research project is to compare the sequences of recently occurring PPRV strains with previously sequenced PPRV isolates in GenBank.

## Methods

### Location

Eighteen states were selected to represent the different agro-ecological zones of the country. These agro-ecological zones were aligned within the six geopolitical zones of the country for ease of reference. At present, there is no comprehensive figure for the population of sheep and goats in Nigeria, but in 1983, a population of 22 million goats and 8 million sheep was estimated (Federal Livestock Department, Lagos, Nigeria). Locations where samples were collected were marked and recorded by the use of a GPS apparatus (GARMIN) GPSMAP 76CSX with sensor.

### Sample collection

Three states were identified randomly from each of the six agro-ecological zones and 20 samples of either oculo-nasal swabs or tissues (lung, spleen and lymph node) were collected with consent of the animals’ owners from each of the 18 states. Swabs were collected from live animals, while tissues were collected from dead animals. The locations for sampling included major towns, their surrounding villages and suburbs. Stratified sampling was used in herd or farm sites, grazing sites, market places and house backyard range. Sampling was carried out during necropsy on dead animals and from sick animals with clinical signs that resembled PPR. The total number of samples collected was 360, which comprised 169 tissue samples and 191 oculo-nasal swabs. To ensure that the quality of the samples was maintained from the field to the laboratory, samples were always kept on iceblocks in a sample collection flask.

### Preparation of necropsy tissues

Necropsy tissues were processed based on the standard operating procedure of the PPR Laboratory, Viral Research Division, National Veterinary Research Institute (NVRI), Nigeria. Briefly, 0.5 g of lung, spleen and lymph node from the same animal were pooled together and ground with a mortar and pestle to which sterile sand and phosphate-buffered saline had been added. The 10% suspension was centrifuged at 1800 g for 5 min and the supernatant filtered through a 0.22-µm filter to remove bacterial and fungal contaminants. Nucleic acid was extracted from the filtrate. The remaining unprocessed tissues were stored at -80 °C for future use.

### Identification of Peste-des-petits-ruminants virus by reverse transcriptase-polymerase chain reaction

A QIAamp Viral RNA Mini extraction kit^®^ Qiagen (Hilden, Germany) was used to extract total RNA from the field samples and from the positive control, according to the manufacturer’s instructions. The positive control was a live attenuated PPRV Nigeria 75/1 vaccine strain produced at the NVRI, Nigeria (Diallo *et al*. 1989; Taylor 1979). The extracted RNA was stored at -80 °C until used.

The samples were screened by a one-step reverse transcriptase–polymerase chain reaction (RT-PCR) assay for the detection of PPRV nucleic acid using a GeneAmp^®^ PCR System (LifeTech) and the following Verso (Thermo-Scientific^®^) protocol: 3.5 µL of ultra-pure sterile water, 1.0 µL RT-Enhancer, 1.0 µL (10 pmole) of each forward primer PPR-NP3 (5’-TCTCGGAAATCGCCTCACAGACTG) and reverse primer PPR-NP4 (5’-CCT CCT CCT GGT CCT GGT CCT CCA GAA TCT), 12.5 µL one-step PCR Hot-Start Master Mix (2×), 1.0 µL of enzyme-mix and 5.0 µL of RNA template in a final volume of 25 µL. The PCR cycling conditions consisted of an initial cycle of 50 °C for 15 min, 95 °C for 15 min, 45 cycles of 95 °C for 30 sec, 55 °C for 30 sec, 72 °C for 30 sec and a final extension at 72 °C for 10 min.

The amplified PCR products were analysed by electrophoresis in a 1% Tris acetate–EDTA agarose gel stained with ethidium bromide at a concentration of 1 µg/mL, and run at 120 V for 45 min. The bands were visualised under ultraviolet light and photographed. A 350-basepair (bp) fragment of the N-gene between nucleotides 1232 and 1583 of the N-gene (Couacy-Hyman *et al*. 2002; EMBL accession no. X74443) was amplified.

### Sequencing

The amplicons were purified using a QIAquick^®^ PCR purification kit (Qiagen^®^, Southern Cross Biotechnology) according to the manufacturer’s instructions, packaged in dry-ice and sent by air from NVRI (Vom, Nigeria) to Inqaba Biotec (South Africa) for sequencing. Amplicons were sequenced directly with a BigDye^®^ Terminator v.3.1 cycle sequencing kit (LifeTech) according to the manufacturer’s instructions. Precipitation of extension products was by the ethanol precipitation protocol of Lifetech^®^. Samples were electrophoresed using the spectruMedix Genetics analysis system SCE 240.

### Phylogenetic analysis

Sequences were assembled using the Staden package (Staden, Beal & Bonfield 2000), aligned with ClustalX v.2.1 (Larkin *et al*. 2007) and edited with BioEdit v.7.2.3 (Hall 1999), and Molecular Evolutionary Genetics Analysis v.5.2. Additional N sequences obtained from PPRV isolated in other endemic countries in the world were obtained from GenBank and were selected based on locations and N-gene lineage.

A 243-nucleotide fragment of the PPRV N-gene (between nucleotides 1307–1549 of sequence GenBank accession number L39878) was used for phylogenetic analysis. A general time-reversible model with a gamma-shaped distribution of rate variation across sites (GTR + G) was selected by Akaike Information Criterion in Modeltest v3.7 (Posada & Crandall 1998). A 1000 bootstrap distance analysis was performed in PAUP* v4b10.

## Results

**FIGURE 1:**
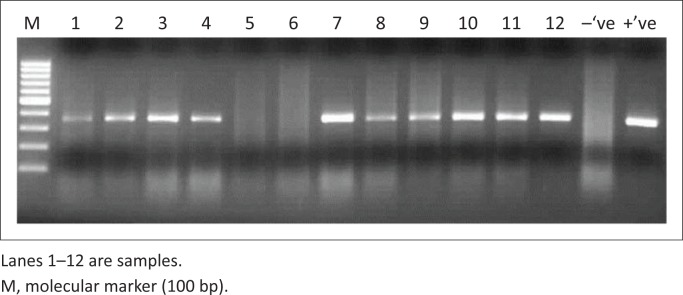
Positive reverse transcriptase–polymerase chain reaction of the peste-des-petits-ruminants virus N-gene of swab/homogenate samples is indicated by a 350-bp band.

Amplicons of the expected 350 bp were obtained by RT-PCR (Figure 1). Eleven of 35 (31.4%) tissue samples from female caprines were positive, 9 of 35 (25.7%) tissue samples from male caprines were positive, 2 of 35 (5.7%) tissue samples from female ovines were positive, 3 of 35 (8.6%) tissue samples from male ovines were positive, 4 of 35 (11.4%) oculo-nasal swabs from female caprine were positive, 5 of 35 (14.3%) oculo-nasal swabs from male caprines were positive, zero of 35 (0%) oculo-nasal swab from female ovines was negative and 1 of 35 (2.9%) oculo-nasal swabs from male ovines was positive for PPRV as indicated in Table 1. A total of 35 samples of 360 (9.7%) tested positive by RT-PCR, of which 25 were from tissue samples and 10 were from oculo-nasal swabs (Table 2). The nucleotide sequences were deposited into GenBank under the accession numbers KF908036–KF908047 (Table 3).

**TABLE 1:** Description of the animals and samples that tested positive for peste-des-petits-ruminants virus by Verso One-Step reverse transcriptase. Tissue samples were pooled.

Sample number	Location	Species	Sex	Age	Sample type	Clinical signs	RT-PCR
003	Langtang-N	Ovine	F	Adult	NS	D, ND	-
005	Jos-North	Caprine	F	Adult	NS	D, ND	-
006	Jos-North	Ovine	M	Adult	NS	D, ND	-
007	Jos-North	Ovine	M	Adult	NS	ND	-
012	Langtang-N	Ovine	F	Adult	L, LN, S	ND	+’ve
014	Bassa	Caprine	F	Adult	L, LN, S	D, ND	+’ve
017	Barakin Ladi	Caprine	F	Adult	L, LN, S	D, ND	+’ve
018	Barakin Ladi	Caprine	F	Adult	L, LN, S	D, ND	+’ve
052	Minna	Caprine	M	Adult	L, LN, S	D, ND	-
053	Minna	Caprine	M	Adult	L, LN, S	ND	+’ve
054	Minna	Caprine	M	Adult	L, LN, S	ND	-
055	Minna	Caprine	M	Adult	L, LN, S	ND	-
058	Bosso	Caprine	F	Adult	L, LN, S	D, ND	+’ve
059	Bosso	Caprine	M	Adult	NS	ND	+’ve
072	Sokoto	Ovine	M	Adult	L, LN, S	ND	+’ve
074	Sokoto	Ovine	M	Adult	L, LN, S	ND	-
079	Sokoto	Caprine	M	Adult	L, LN, S	ND	+’ve
082	Kano	Caprine	M	Adult	NS	ND	+’ve
096	Kano	Caprine	M	Adult	L, LN, S	D, ND	+’ve
114	Kaduna	Caprine	F	Adult	L, LN, S	D, ND	+’ve
117	Tudun Wada	Ovine	F	Adult	L, LN, S	ND	-
118	Tudun Wada	Caprine	F	Adult	L, LN, S	ND	+’ve
119	Tudun Wada	Caprine	M	Adult	L, LN, S	ND	+’ve
144	Yola	Caprine	F	Adult	NS	ND	+’ve
151	Yola	Caprine	M	Adult	L, LN, S	D, ND	+’ve
152	Yola	Caprine	F	Adult	L, LN, S	D, ND	+’ve
153	Yola	Caprine	M	Adult	L, LN, S	ND	-
155	Yola	Caprine	M	Adult	L, LN, S	ND	-
156	Yola	Caprine	M	Adult	L, LN, S	D, ND	-
162	Bauchi	Caprine	F	Adult	NS	ND	-
163	Bauchi	Caprine	M	Adult	NS	ND	+’ve
170	Bauchi	Caprine	M	Adult	NS	ND	+’ve
175	Bauchi	Ovine	F	Adult	L, LN, S	D, ND	-
177	Bauchi	Caprine	F	Adult	L, LN, S	D, ND	+’ve
184	Agege	Caprine	M	Adult	NS	ND	+’ve
185	Agege	Caprine	F	Adult	NS	ND	+’ve
187	Agege	Ovine	F	Adult	NS	ND	-
188	Agege	Ovine	F	Adult	NS	D, ND	-
192	Agege	Ovine	F	Adult	L, LN, S	D, ND	+’ve
205	Ibadan-N	Caprine	F	Adult	NS	ND	+’ve
207	Ibadan-N	Ovine	M	Adult	NS	D, ND	+’ve
211	Ibadan-N	Caprine	M	Adult	L, LN, S	D, ND	+’ve
213	Ibadan-N	Caprine	M	Adult	L, LN, S	D, ND	+’ve
218	Ibadan-N	Ovine	M	Adult	L, LN, S	D, ND	+’ve
219	Ibadan-N	Ovine	M	Adult	L, LN, S	D, ND	+’ve
220	Ibadan-N	Caprine	M	Adult	L, LN, S	D, ND	-
230	Oje-Oba	Caprine	M	Adult	NS	ND	+’ve
238	Akure-South	Caprine	F	Adult	L, LN, S	ND	+’ve
239	Akure-South	Caprine	M	Adult	L, LN, S	ND	-
305	Ikot-Ebom	Caprine	F	Adult	NS	ND	+’ve
310	Etaha-Itan	Caprine	F	Adult	NS	D, ND	+’ve
316	Etaha-Itan	Caprine	F	Adult	L, LN, S	D, ND	+’ve
320	Etaha-Itan	Caprine	F	Adult	L, LN, S	ND	+’ve

NS, nasal swab; L, lung; LN, lymph node; S, spleen; D, Clinical signs included diarrhoea; ND, nasal discharge; RT-PCR, peste-des-petits-ruminants virus by Verso One-Step reverse transcriptase.

**TABLE 2:** Description of the regions, study areas and period of sample collection, location and numerical number of positives in percentage and total number of samples collected.

Region	State	Location	Tissue		Swab		Total	
		
			Number positive/number collected	%	Number positive/number collected	%	Number positive/number collected	%
North-central (July 2010)	Plateau	Langtang-N	1/11	9	0/3	0	1/14	7
		Barakin Ladi	2/4	50	0/1	0	2/5	40
		Jos-North	-	-	0/3	0	0/3	0
		Bassa	1/4	25	0/2	0	1/6	17
	Benue	Makurdi	0/8	0	0/11	0	0/19	0
		Gboko	0/1	0	0/0	0	0/1	0
	Niger	Minna	1/1	100	0/10	0	1/11	9
		Bosso	1/9	22	1/0	0	2/9	22
	**Total**	**-**	**7/38**	**18**	**0/30**	**0**	**7/68**	**10**
North-west (August 2010)	Sokoto	Sokoto	2/10	20	0/10	0	2/20	10
	Kano	Kano	1/10	10	1/10	10	2/20	10
	Kaduna	Zango	0/0	0	0/10	0	0/10	0
		Tudun Wada	3/10	30	0/0	0	3/10	30
	**Total**	**-**	**6/30**	**20**	**1/30**	**3**	**7/60**	**12**
North-east (September 2010)	Borno	Maiduguri	0/10	0	0/10	0	0/20	0
	Adamawa	Yola	2/12	17	1/8	13	3/20	15
	Bauchi	Bauchi	1/10	10	2/10	20	3/20	15
	**Total**	**-**	**3/32**	**9**	**3/28**	**10**	**6/60**	**10**
South-west (October 2010)	Lagos	Agege	1/11	9	2/9	22	3/20	15
	Oyo	Ibadan	4/10	40	2/10	20	6/20	30
	Ondo	Oje-Oba	1/3	33	0/11	0	1/14	7
		Akure-South	1/6	17	0/0	0	1/6	17
	**Total**	**-**	**7/30**	**23**	**4/30**	**13**	**11/60**	**18**
South-east (November 2010)	Abia	Okwoyi	0/0	0	0/6	0	0/6	0
		Umuahia	0/6	0	0/8	0	0/14	0
	Imo	Mbaitoli	0/0	0	0/11	0	0/11	0
		Afor-Nnobi	0/4	0	0/0	0	0/4	0
		Orodo	0/5	0	0/0	0	0/5	0
	Enugu	Emenne	0/10	0	0/10	0	0/20	0
	**Total**	**-**	**0/25**	**0**	**0/35**	**0**	**0/60**	**0**
South-south (January 2011)	Akwa-Ibom	Ikot-Ebom	0/0	0	1/3	30	1/3	30
		Etaha-Itan	2/9	22	1/8	13	3/17	18
	Cross-Rivers	Akamkpa	0/0	0	0/7	0	0/7	0
		Calabar	0/9	0	0/4	0	0/13	0
	Bayelsa	Sagbama	0/4	0	0/16	0	0/20	0
	**Total**	**-**	**2/22**	**9**	**2/38**	**5**	**3/60**	**5**

**Total**	**-**	**-**	**25/169**	**15**	**10/191**	**5**	**35/360**	**10**

**TABLE 3:** National Center for Biotechnology Information**** GenBank accession numbers of sequences described in this study.

Number Sequence name	Accession number
1. NG 0Y 2013 00213*	KF908036
2. NG PL 2010 00018*	KF908037
3. NG SO 2010 00079	KF908037
4. NG OY 2013 00205	KF908037
5. NG ON 2010 00230*	KF908038
6. NG VACNVRI75/1	KF908038
7. NG SO 2010 00072*	KF908039
8. NG PL 2010 00014	KF908039
9. NG PL 2010 00017	KF908039
10. NG BA 2013 00177	KF908039
11. NG OY 2013 00207	KF908039
12. NG KN 2010 00096*	KF908040
13. NG AD 2010 00151	KF908040
14. NG BA 2013 00170*	KF908041
15. NG KD 2010 00119	KF908041
16. NG LA 2010 00184*	KF908042
17. NG KD 2010 00118	KF908042
18. NG AD 2010 00152	KF908042
19. NG BA 2010 00163	KF908042
20. NG NI 2010 00058*	KF908043
21. NG OY 2013 00211*	KF908044
22. NG PL 2010 00012*	KF908045
23. NG OY 2010 00218*	KF908046
24. NG LA 2010 00185	KF908046
25. NG LA 2010 00192	KF908046
26. NG OY 2010 00219	KF908046
27. NG AK 2010 00316	KF908046
28. NG AK 2013 00320	KF908046
29. NG AK 2013 00310*	KF908047

Sequences without an asterisk (*) indicate those sequences that were identical to another sequence submitted to GenBank.

**FIGURE 2:**
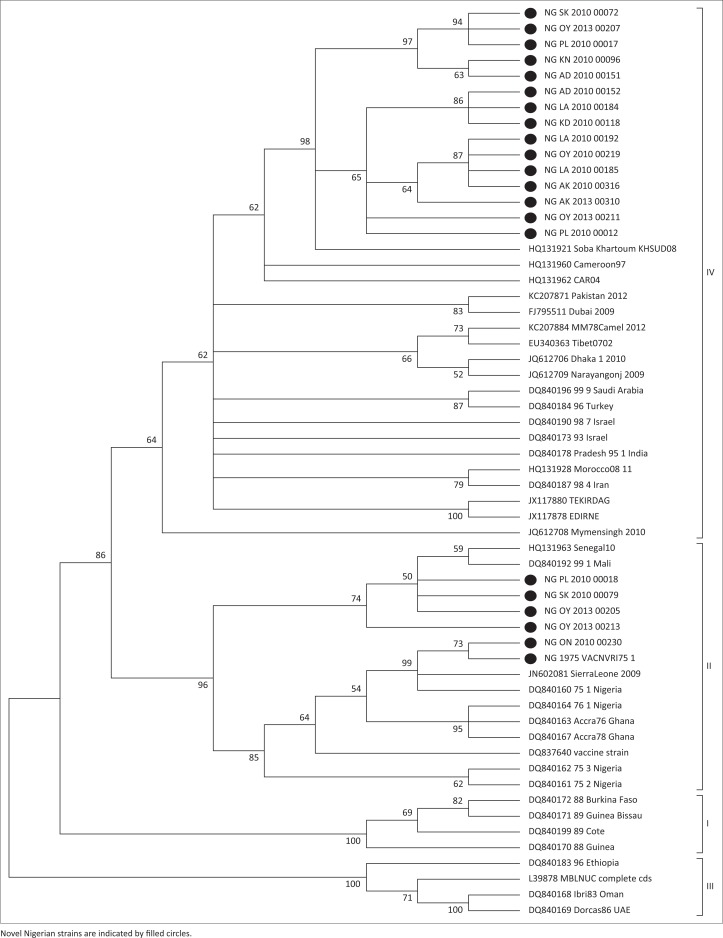
Fifty percent majority-rule consensus cladogram of partial unrooted peste-des-petits-ruminants virus N-gene sequences from this study and GenBank. The phylogeny was determined by distance analysis in Phylogenetic Analysis Using Parsimony.

An unrooted bootstrapped 50% majority-rule consensus cladogram was produced. The sequences from Oman Ibri and Ethiopia of lineage III grouped together (bootstrap value 100%). Sequences from Côte d’Ivoire, Senegal and Burkina Faso grouped together in lineage I (bootstrap value 100%). The next clade consisted of Nigeria 75/1, Ghana 78 and Mali sequences in lineage II (bootstrap value 97%). The tree branched to lineage IV, which consisted of Indian and Israeli sequences (bootstrap value 88%) (Figure 2).

The genetic distances, mean and standard deviation calculated between the Nigerian PPRV strains and the GenBank Nigerian strains are summarised in Table 4.

**TABLE 4:** Genetic distances between peste-des-petits-ruminants virus N GenBank Nigerian sequences and the recent Nigerian sequences, using a general time-reversible model with a gamma-shaped distribution of rate variation across sites (GTR + G).

Number	Nigerian sequences	NG VACNVRI751	DQ840160 75 1Nigeria	DQ840161 75 2 Nigeria	DQ840162 75 3 Nigeria	DQ840164 76 1 Nigeria	Mean ± standard deviation
1	NG OY 2013 00213	0.0745	0.069	0.0479	0.0523	0.0674	0.0062 ± 0.0115
2	NG PL 2010 00018	0.0805	0.0748	0.0587	0.0633	0.0633	0.0068 ± 0.0091
3	NG SO 2010 00079	0.0805	0.0748	0.0587	0.0633	0.0633	0.0068 ± 0.0091
4	NG OY 2013 00205	0.0805	0.0748	0.0589	0.0633	0.0633	0.0068 ± 0.0091
5	NG ON 2010 00230	-	0.0415	0.0398	0.0443	0.0004	0.0032 ± 0.0187
6	NG VACNVRI751	-	0.0415	0.0398	0.0443	0.0004	0.0032 ± 0.0187
7	NG PL 2010 00017	0.0198	0.0189	0.0164	0.0169	0.0185	0.0181 ± 0.0142
8	NG SO 2010 00072	0.0198	0.0189	0.0164	0.0169	0.0185	0.0181 ± 0.0142
9	NG PL 2010 00014	0.0198	0.0189	0.0164	0.0169	0.0185	0.0181 ± 0.0142
10	NG BA 2013 00177	0.0198	0.0189	0.0164	0.0169	0.0185	0.0181 ± 0.0142
11	NG OY 2013 00207	0.0198	0.0189	0.0164	0.0169	0.0185	0.0181 ± 0.0142
12	NG KN 2010 00096	0.0183	0.0175	0.0015	0.0156	0.0172	0.0167 ± 0.0137
13	NG AD 2010 00151	0.0183	0.0175	0.0015	0.0156	0.0172	0.0167 ± 0.0137
14	NG KD 2010 00119	0.0196	0.0188	0.0174	0.0018	0.0196	0.0187 ± 0.0097
15	NG BA 2013 00170	0.0196	0.0188	0.0174	0.0018	0.0196	0.0187 ± 0.0097
16	NG LA 2010 00184	0.0018	0.0172	0.0147	0.0152	0.0168	-
17	NG KD 2010 00118	0.0018	0.0172	0.0147	0.0152	0.0168	0.0164 ± 0.0137
18	NG AD 2010 00152	0.0018	0.0172	0.0147	0.0152	0.0168	0.0164 ± 0.0137
19	NG BA 2010 00163	0.0018	0.0172	0.0147	0.0152	0.0168	0.0164 ± 0.0137
20	NG NI 2010 00058	0.0173	0.0165	0.0014	0.0146	0.0161	0.0157 ± 0.0135
21	NG OY 2013 00211	0.0186	0.0178	0.0153	0.0158	0.0174	0.0170 ± 0.0140
22	NG PL 2010 00012	0.0173	0.0165	0.0014	0.0146	0.0161	0.0157 ± 0.0135
23	NG OY 2010 00218	0.0183	0.0175	0.0015	0.0156	0.0171	0.0167 ± 0.0137
24	NG AK 2013 00320	0.0183	0.0175	0.0015	0.0156	0.0171	0.0167 ± 0.0137
25	NG LA 2010 00185	0.0183	0.0175	0.0015	0.0156	0.0171	0.0167 ± 0.0137
26	NG LA 2010 00192	0.0183	0.0175	0.0015	0.0156	0.0171	0.0167 ± 0.0137
27	NG AK 2010 00316	0.0183	0.0175	0.0015	0.0156	0.0171	0.0167 ± 0.0137
28	NG OY 2010 00219	0.0183	0.0175	0.0015	0.0156	0.0171	0.0167 ± 0.0137
29	NG AK 2013 00310	0.0184	0.0176	0.0151	0.0157	0.0172	0.0168 ± 0.0138
30	HQ131921 Soba Khartoum KHSUD08	0.0165	0.0157	0.0133	0.0139	0.0154	0.0150 ± 0.0131
31	KC207871 Pakistan 2012	0.0202	0.0193	0.0167	0.0016	0.0189	0.0182 ± 0.0178
32	DQ840170 88 Guinea	0.0235	0.0226	0.0213	0.0206	0.0235	0.0223 ± 0.0131
33	DQ840169 Dorcas86 UAE	0.0308	0.0297	0.0281	0.0288	0.0306	0.0296 ± 0.0115

## Discussion

The aim of this study was to ascertain whether there were genetic variations between the present strains and previously isolated strains circulating in the country and to determine the geographical lineage(s) of the studied PPRV.

PPR was also known in the past in Nigeria as ‘Kata’ and goat plaque all over the world. In most developing countries including Nigeria, small-ruminant farming covers the basic necessities of the majority of peasants. Sheep and goats are essential sources of subsistence. They improve the survival of the poor people in these countries. Unfortunately, PPR is adversely threatening the production of sheep and goats in endemic areas. PPR is a trans-boundary disease and is part of the group of economically important animal diseases whose outbreaks should be notified to the World Organisation for Animal Health (OIE for International des Epizooties). In Nigeria, because of the high importance of sheep and goats for poor farmers, the control of diseases that have a negative impact on their production is a major goal for programmes aimed at poverty alleviation. Sheep and goats are reared in the same fashion in Nigeria with slight differences between the savannah north and tropical rain forest zone of the south.

The animals are rarely housed or tethered, except in areas such as eastern Nigeria where pressure on land is creating competition between crops and livestock, so the latter must be restrained (Carew 1982; Sempeho 1982). The animals roam freely, requiring minimum investment in housing and feeds, non-labour intensive and are efficient in the utilisation of roughages, farm residues and agro-industrial by-products. In Nigeria, sheep and goats are widely distributed all over the country across diverse ecological and climatic zones, with the pattern of ownership cutting across socio-economic status.

In Nigeria, it has been estimated that sheep and goats provide over 35% of the total animal protein consumed. Their hides support the leather industry and at a conservative off-take value of 600 naira for a small ruminant, the total financial value of the small-ruminant industry in Nigeria could be put at about 40 billion naira, based on an estimated population of 34.45 million goats and 22.09 million sheep (Shamaki 2002). However, disease remains a major limiting factor in their production. The diseases that cause significant deaths in sheep and goats include bacterial and viral pneumonia, parasitic and viral gastro-enteritis, ectoparasitism as well as nutritional and metabolic diseases. Among these, PPR is of paramount significance as a disease entity, as it causes both respiratory and gastro-intestinal tract disorders.

The expected number of 60 samples per zone and 1 PPRV amplicon sequence representation per state with location of sampling mapped by GPS coordinates was achieved across the country except for the riverine Niger Delta of Bayelsa state where movement to villages was difficult because of lack of access roads, and northern states because of the insurgence of the Boko Haram militia. However, sampling was stratified and targeted sheep and goats with classical or sub-clinical signs of fever and depression, ocular and nasal discharges, diarrhoea and emaciation. The sampling was more of a passive surveillance in locations within the study areas, which included the six agro-ecological zones of Nigeria, as indicated in Table 1.

The 35 positive samples out of a total of 360 (9.7%) samples were from all the agro-ecological zones except the south-eastern zone for which no positive results were obtained by RT-PCR, although some farmers in the zone complained of losing all their goats to an epidemic that resembled PPR prior to sampling. Livestock are not allowed to roam freely in this region, thereby limiting the chances of cross-transmission of the disease from one flock to another during an outbreak. Other possible reasons for the negative results in this area could be because of very low viral load, which could not be detected by the assay, or it could be that the RNA was lost during processing. Previous findings by Wosu *et al*. (1994) and Obi *et al*. (1983) revealed that PPR was prevalent in the south-east zone of the country.

Based on the results in Tables 1 and 2 and the percentages of the analysed results according to species, sex and breed, it was concluded that PPRV in an infected population affects both sheep and goats, male and female animals of both species, and equally affects both the long-legged species of the north and the short-legged species or West African dwarf goats or sheep of the south.

Fourteen samples collected from the study areas of the north-central region in the month of July, 9 samples from the north-west collected in August, 11 samples from the north-east collected in September, 15 samples from the south-west collected in October, 3 samples from the south-south collected in January tested positive by RT-PCR for PPRV, while none collected from south-east in November tested positive. These findings suggest that PPR was prevalent in Nigeria during the sampled period. However, a study conducted by Wosu *et al*. (1990) reported that PPR incidence was higher in south-eastern Nigeria during the dry Hamattan season (December – January) than in the rainy season with a peak in April. Sampling before the expected period of high incidence of PPR outbreaks in the south-eastern region could also be the reason for the inability to get positive samples in this zone.

Cattaneo *et al*. (1987) suggested that the N-gene of PPRV was more abundant in positive tissue samples and Couacy-Hymann *et al*. (2002) used the N-gene to detect PPRV in suspected samples. Targeting of PPRV N-gene for PCR gave very promising results. N-gene codes for an internal structural protein and mRNAs of the N-gene are the most abundant transcripts of the virus, making it an attractive target for development of a highly sensitive and specific diagnostic assay for PPRV (George 2002). Based on clinical signs and the detection of PPRV using RT-PCR, it was concluded that the disease outbreaks in the present study areas were caused by PPRV, although tests for other pathogens were not carried out.

The genetic similarity and divergence among the present PPRV sequence strains of Nigeria and their relatedness to some of the previously described isolates in GenBank were analysed. The NCBI BLAST search revealed that some Nigerian PPRVs obtained in this study had an identity of 100% for Nigeria 75/1 and 95% for Nigeria 76/1, and unexpectedly most of the strains recently obtained had 97% – 99% identity to strains from the Republic of Gabon (97%) and (96%) identity to strains from the Republic of Iran (96%) (Table 3).

Nucleotide genetic diversity was examined (Table 4), and the shortest genetic mean distance ± standard deviation (0.032 ± 0.0187) was between the recent PPRV strains of lineage II NG VACNVRI 75/1. This suggests that of the present PPR vaccine produced in NVRI, Vom is similar to the previous Nigerian strains deposited in GenBank. Even though the vaccine viral strain passed through a series of tissue culture attenuations, the genetic relationship between the field strain Nigeria 75/1 and the vaccine strain NG VACNVRI 75/1 is still similar, that is, using the (GTR + G), the genetic distance is 0.00415, the mean ± standard deviation is 0.032 ± 0.0187 which are significantly negligible. The longest mean genetic distance (0.0681 and 0.00913) was between lineage II samples from Plateau, Sokoto and Oyo states. This increase in genetic distance could be because of genetic drift over time. In lineage IV of the recently studied strains, the shortest genetic mean distance (0.0157) was from the Niger and Plateau states, and the longest genetic mean distance variable (0.0187) was from Kaduna and Bauchi states. The emergence of these new strains in Nigeria is probably because of the introduction of the strains from other lineage IV PPRV-endemic countries through trade.

Based on previous studies of geographical distribution of PPRV (Couacy-Hymann *et al*. 2002; Dhar *et al*. 2002; Ozkul *et al*. 2002; Shaila *et al*. 1996), all Nigerian strains/isolates normally clustered together into a separate branch from all the Asian isolates. Unexpectedly, the new Nigerian strains clustered in two separate lineages (Figure 2). Strains from Sokoto, Oyo and Plateau states clustered with existing strains from Mali-91 (DQ840192), Senegal-2010 (HQ131963) and Sierra-Leone-2009 (JN802080) of lineage II. Recent strains from Ondo state and the NVRI Vaccine 75/1 isolate clustered with the previous Nigeria 75/1 (DQ840160), Nigeria 75/2 (DQ840161), Nigeria 75/3 (DQ840162) and Ghana 78 (DQ840166) of lineage II. In lineage IV, new strains from Plateau, Bauchi, Oyo, Sokoto, Kano, Adamawa, Kaduna and Bauchi states formed a sub-cluster, and strains from Adamawa, Bauchi, Lagos, Kaduna, Plateau, Niger, Oyo and Akwa-Ibom formed another sub-cluster. The strain from Sudan 2008 (HQ131921) was closely related to the new Nigerian strains compared to other GenBank isolates in lineage IV.

**FIGURE 3:**
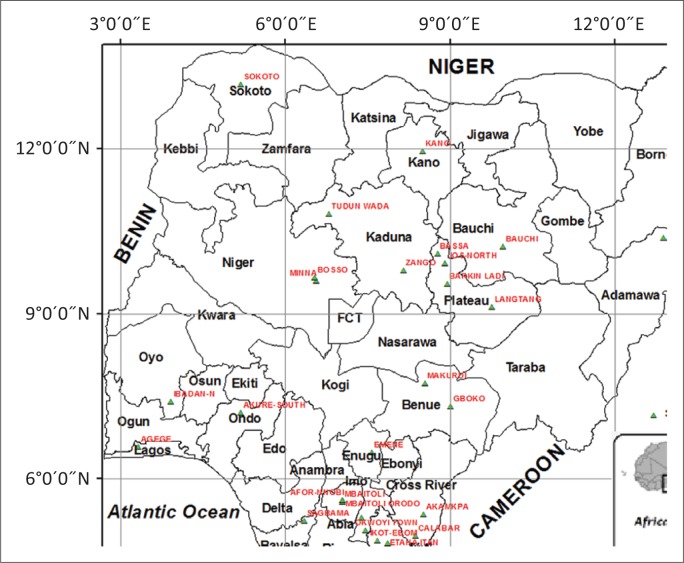
Map of Nigeria describing the GPS location of sampling indicated in red ink and lineages isolated.

This finding suggests that both lineage II and IV strains of PPRVs are circulating presently in all the studied agro-ecological zones of Nigeria as indicated in Figure 3 and as described by Woma *et al*. (2015). Despite the fact that the recent strains from lineage IV seem to predominate in most of the study area, there is no clear demarcation of the state or region where these new strains are found. They appear to be spread all over the study area as indicated in Figure 3. The introduction of the new strain is probably because of the movement of sheep and goats from the Sahel African countries into Nigeria and from the northern dry savannah region to the wet forest southern region of the country for grazing during the dry season. Trade of animals and animal products from other African countries to Nigeria and from one state to another may serve as the major source of transmission of trans-boundary animal diseases including PPRV. The presence of the new strain of PPRV in the country is not surprising because Nigeria is a major hub of animal product consumption in West Africa because of its large population. Meeting the ever-increasing domestic demand leads to the importation of sheep and goats from the neighbouring Sahel countries that raise livestock. Low disease monitoring, introduction and re-introduction of many exotic diseases and disease strains have been traced to the animals that entered the country through the porous borders.

The data obtained from this study have provided important and additional information on the molecular epidemiology of PPR virus in Nigeria. However, the clustering of sequences in different areas indicates that the PPRV population in Nigeria is more diverse than previously thought and this study provides a better epidemiological picture of PPRV in endemic areas of the world, such as Nigeria.

## Conclusion

The results obtained from this study suggest that PPR still remains endemic with sporadic epidemics in Nigeria and other sub-Saharan African countries. Regular outbreaks in areas where it was considered exotic in the past are conceivably because of an increase in trade and commerce. During this study, molecular characterisation of PPR viruses based on the amplification and sequencing of fragments of the N-gene showed that the PPRV from Nigeria belonged to two different lineages: II and IV, with two sub-clusters in lineage II and two sub-clusters in lineage IV. The new strains were related most closely to isolates from the East African countries of Gabon, Central Republic of Africa and Sudan via Cameroon and Chad. However, there was no clear demarcation that separated where these two different strains were found in Nigeria, but rather both appeared to be found in all the study areas. This suggests that both strains are endemic and circulating all over the country.

Considering the wide distribution of PPR in the world, it would be very helpful to institute an investigative and monitory measure to check the movement of animals from one location to another, especially for countries where PPR is still an exotic disease. PPR should be included in the list of differential diagnoses for pneumo-enteritis in small ruminants in non-endemic countries, and sero-surveillance for PPR in countries bordering endemic and affected countries especially in southern Africa, should be considered.
